# Seroepidemiological Study of Parvovirus B19 Infection Among Pregnant Women Attending Ayatollah Rohani Hospital in Babol, Iran: A Cross‐Sectional Study

**DOI:** 10.1002/hsr2.71474

**Published:** 2025-11-09

**Authors:** Zeinab Darbandi, Amir Mohsenfar, Arefeh Ebrahimian Shiadeh, Farzane Sadeghi, Soheil Ebrahimpour, Hemmat Gholinia, Maryam Javadian, Masoumeh Golchoub, Farzin Sadeghi, Masomeh Bayani

**Affiliations:** ^1^ Student Research Committee Babol University of Medical Sciences Babol Iran; ^2^ Department of Medical Microbiology, Faculty of Medicine Golestan University of Medical Science Gorgan Iran; ^3^ Department of Gynecology and Obstetrics, School of Medicine Babol University of Medical Sciences Babol Iran; ^4^ Infectious Diseases and Tropical Medicine Research Center, Health Research Institute Babol University of Medical Sciences Babol Iran; ^5^ Social Determinants of Health Research Center, Health Research Institute Babol University of Medical Sciences Babol Iran; ^6^ Cellular and Molecular Biology Research Center, Health Research Institute Babol University of Medical Sciences Babol Iran

**Keywords:** ELISA, fetal complications, IgG, IgM, Iran, maternal infection, parvovirus B19, pregnancy, seroprevalence, vertical transmission

## Abstract

**Background and Aims:**

Parvovirus B19 is a common viral pathogen associated with a variety of clinical manifestations, including significant complications during pregnancy such as fetal anemia, miscarriage, and hydrops fetalis. Determining the seroprevalence of B19‐specific antibodies in pregnant women is essential for assessing population immunity and potential risk to the fetus. This study aimed to evaluate the seroprevalence of IgG and IgM antibodies against parvovirus B19 among pregnant women attending Ayatollah Rouhani Hospital in Babol, Iran.

**Methods:**

A cross‐sectional study was conducted on 247 pregnant women in 2018. Serum samples were analyzed for parvovirus B19‐specific IgG and IgM antibodies using enzyme‐linked immunosorbent assay (ELISA). Associations between seropositivity and demographic/clinical variables were statistically analyzed.

**Results:**

Out of the 247 participants, 124 (50%) were seropositive for B19‐specific IgG, indicating previous exposure. Additionally, 10 of 90 women (11.1%) tested positive for IgM antibodies, suggesting recent infection. No statistically significant associations were found between seropositivity and variables such as age, gestational age, or history of miscarriage in the multivariate analysis, although parity showed significance in the univariate analysis.

**Conclusion:**

Approximately half of the studied pregnant women had no detectable immunity to parvovirus B19 and were at risk of primary infection during pregnancy. The presence of IgM antibodies in a subset of women highlights the need for increased awareness, routine screening, and preventive strategies to mitigate the risk of vertical transmission and adverse fetal outcomes.

## 1 Introduction

Parvovirus B19 (B19V), the only known pathogenic human parvovirus, is most widely recognized as the causative agent of erythema infectiosum, a common childhood illness [1]. However, its clinical implications extend beyond pediatrics, particularly in the context of pregnancy [2]. B19V infection during gestation poses significant risks to fetal health due to its predilection for erythroid progenitor cells in the fetal liver, the principal site of hematopoiesis during early development [3]. Vertical transmission occurs in approximately one‐third to one‐half of maternal infections and may result in serious fetal outcomes, including miscarriage, hydrops fetalis, and intrauterine fetal demise [4]. Notably, the second trimester represents the period of highest vulnerability for adverse fetal consequences [5]. Despite the widespread nature of B19V and the fact that 50%–75% of women of reproductive age demonstrate serologic evidence of prior exposure, the infection often remains asymptomatic in pregnant women, making timely diagnosis challenging [6]. Given the risk of fetal complications and the asymptomatic presentation of many maternal infections, accurate and early detection of B19V is crucial in prenatal care [7]. Serological testing for anti‐B19 IgM and IgG antibodies remains the primary diagnostic approach [7]. B19V IgM, detectable from 8 to 12 days after infection, confirms recent infection and typically persists for 3 to 6 months. In contrast, IgG antibodies appear later and indicate past exposure and possible long‐term immunity [8]. Despite the clinical relevance of B19V infection in pregnancy, there is a paucity of data on its seroprevalence in various populations, including in Iran [9, 10, 11]. Several studies have evaluated the seroprevalence of human parvovirus B19 (B19V) in different populations across Iran. A recent meta‐analysis by Moosazadeh et al. reported an overall seroprevalence of 54.9% among the general population, with regional variations attributed to socioeconomic and climatic factors [12]. In blood donors, B19V IgG seropositivity rates have been reported between 30% and 60%, reflecting cumulative exposure to the virus over time [13]. Regarding pregnant women, previous studies have shown a seroprevalence of 44.1% in Kerman [9], 55.7% in Ahvaz [14], and 69.1% in Ardabil [15]. These findings suggest that a considerable proportion of Iranian women of reproductive age may still be susceptible to primary B19V infection during pregnancy, which underscores the importance of continued surveillance and preventive strategies. This study aims to assess the seroprevalence of anti‐B19V IgG and IgM antibodies in pregnant women in Babol, Northern Iran, to provide valuable insights into the presence of individuals who are susceptible to infection (i.e., those lacking protective antibodies).

## 2 Methodology

### 2.1 Study Design

This cross‐sectional study was conducted on pregnant women who attended the obstetric clinic at Ayatollah Rouhani Hospital for routine prenatal examinations between March and August 2018. Women presenting with clinical symptoms suggestive of acute parvovirus B19 infection were not specifically targeted for inclusion. The primary objective was to assess the rate of previous and recent infections by detecting the presence of specific antibodies, thus identifying women at risk of fetal complications due to vertical transmission. Participation was limited to pregnant women who provided written informed consent after receiving a full explanation of the study's purpose and protocol. Women who declined to participate were excluded from the study.

### 2.2 Study Population and Sampling

The study population consisted of pregnant women who presented to the obstetrics clinic at Ayatollah Rouhani Hospital. A census sampling approach was employed, in which all eligible participants during the defined period were included. Initially, 250 pregnant women were recruited; however, three individuals were excluded due to incomplete data, resulting in a final sample size of 247 participants. The sample size was estimated using the standard formula for prevalence studies: *n* = *Z*² × *p* × (1 − *p*)/*d*², where *Z* = 1.96 (corresponding to a 95% confidence level), *p* = 0.75 (expected prevalence), and *d* = 0.05 (precision or margin of error). The expected prevalence of 75% was selected based on previous reports of parvovirus B19 IgG seropositivity among pregnant women in Iran, particularly the study by Habibzadeh et al., which reported a prevalence of 69.1%, and a national meta‐analysis by Moosazadeh et al., which found rates ranging from 44.1% to 75% across different regions. Based on these values, the minimum estimated sample size was 288 participants. However, due to limitations in time and available resources, 247 participants were ultimately enrolled in the study [12, 15].

### 2.3 Data Collection

Blood samples were collected from all participants during routine prenatal visits. After centrifugation, serum samples were aliquoted and stored at –20°C in the virology laboratory of the Department of Microbiology, Babol University of Medical Sciences. The detection of IgG and IgM antibodies specific to parvovirus B19 was performed using a commercial enzyme‐linked immunosorbent assay (ELISA) kit (Euroimmun Anti‐Parvovirus B19 ELISA, Germany).

### 2.4 Laboratory Protocols

The assay was carried out in accordance with the manufacturer's instructions, which involved incubation of patient sera in microplates coated with recombinant viral antigens, followed by multiple washing steps to remove unbound antibodies. A horseradish peroxidase‐conjugated secondary antibody was added, and after further incubation and washing, a substrate solution containing tetramethylbenzidine and hydrogen peroxide was introduced. The enzymatic reaction was terminated using sulfuric acid, and the optical density of each well was measured using an ELISA reader. Antibody concentrations were calculated based on calibrator values provided in the kit and expressed in international units per milliliter (IU/mL).

### 2.5 Variables and Measures

The main outcome variables included the presence and quantitative level of IgG antibodies against parvovirus B19, measured in IU/mL using a commercial ELISA kit (Euroimmun, Germany). IgG concentrations were calculated based on calibrator standards provided by the manufacturer, following the kit's instructions for quantitative interpretation. Additionally, the presence of IgM antibodies was assessed qualitatively and recorded as positive, negative, or borderline. Secondary data collected included maternal age, gestational week and trimester, parity, body mass index (BMI), educational status, and place of residence (urban or rural). These variables were obtained through patient records and interviews and were categorized appropriately for analysis.

### 2.6 Data Analysis

Data analysis was performed using SPSS software, version 22.0 (IBM Corp., Armonk, NY, USA). Descriptive statistics (mean, standard deviation [SD], and percentages) were used to summarize demographic and serological data. Associations between categorical variables and serostatus were analyzed using Chi‐square tests, and non‐parametric tests (Mann–Whitney *U*‐test and Kruskal–Wallis test) were used to compare IgG antibody levels across groups. To identify independent predictors of IgG seropositivity, multivariable logistic regression analysis was performed. Odds ratios with 95% confidence intervals were reported. All statistical tests were two‐sided, and the a priori level of significance was set at *α* = 0.05. Analyses were based on pre‐specified hypotheses, and no post hoc subgroup analyses were performed. Statistical reporting followed the SAMPL guidelines (Statistical Analyses and Methods in the Published Literature) and the recommendations of Assel et al. (16) for clinical research reporting.

### 2.7 Ethical Considerations

Ethical approval for this study was obtained from the Ethics Committee of Babol University of Medical Sciences (IR.MUBABOL.HRI.REC.1398.205). All participants received a complete description of the study and signed informed consent forms before sample collection. To maintain confidentiality, all data were anonymized and securely stored. All procedures in this study were conducted in accordance with the ethical standards of the institutional research committee and the Declaration of Helsinki. The methodology was designed and described in sufficient detail to enable replication by other researchers. The use of validated commercial kits, standard laboratory protocols, and comprehensive data analysis ensured the reliability and reproducibility of the study's findings.

## 3 Results

### 3.1 Seroprevalence of Parvovirus B19 Antibodies

Out of 247 participants, 124 (50.2%) tested positive for IgG antibodies against parvovirus B19, indicating past exposure, while 10 of 90 women (11.2%) tested positive for IgM antibodies, suggestive of recent infection. Five participants (2.0%) showed borderline IgG results, and 5 (5.6%) showed borderline IgM results. Among the 10 IgM‐positive cases, 4 (40%) were also IgG‐positive, whereas 6 (60%) were IgG‐negative. Table 1 shows the serological profile of parvovirus B19 antibodies in pregnant women, presenting data on the IgG and IgM antibody status in their blood samples.

**Table 1:** Serological profile of parvovirus B19 antibodies in pregnant women.

Antibody type	Result	Number of cases (*n*)	Percentage (%)	Total sample tested
IgG	Positive	124	50.2	247
	Negative	118	47.8	
	Borderline	5	2.0	
IgM*	Positive	10	11.2	90
	Negative	75	83.3	
	Borderline	5	5.5	

* IgM testing was performed on a subset of 90 pregnant women due to kit limitations.

### 3.2 Demographic and Clinical Characteristics

The mean age of participants was 28.06 ± 5.29 years (range: 17–42 years). The distribution of participants by age group is as follows: < 18 years (2%), 19–34 years (86.6%), and ≥ 35 years (11.3%). The mean gestational age was 18.55 ± 8.60 weeks (range: 6–38 weeks). No statistically significant difference was observed between the mean age of IgG‐positive (28.24 ± 5.58 years) and IgG‐negative participants (27.87 ± 4.98 years) (*p* = 0.58).

### 3.3 Association Between IgG Antibody Status and Sociodemographic and Clinical Variables

Statistical analysis revealed no significant correlation between IgG antibody positivity and variables such as age group, education level, place of residence, BMI, gestational age, pregnancy order, or trimester (all *p* > 0.05). Table 2 shows the relationship between IgG seropositivity and selected sociodemographic and clinical variables.

**Table 2:** Association between IgG antibody status and sociodemographic/clinical variables (Chi‐square test).

Variables	Total, *n* (%)	IgG negative, *n* (%)	IgG positive, *n* (%)	Borderline, *n* (%)	*p* value*
Age group (years)					
≤ 30	153 (61.9%)	74 (48.4%)	75 (49.0%)	4 (2.6%)	0.65
> 30	94 (38.1%)	44 (46.8%)	49 (52.1%)	1 (1.1%)	
Education level					
Illiterate	169 (68.4%)	77 (45.6%)	89 (52.7%)	3 (1.8%)	0.50
Literate	78 (31.6%)	41 (52.6%)	35 (44.9%)	2 (2.6%)	
Place of residence					
Urban	98 (39.7%)	44 (44.9%)	52 (53.1%)	2 (2.0%)	0.76
Rural	149 (60.3%)	74 (49.7%)	72 (48.3%)	3 (2.0%)	
BMI					
Normal	37 (15.0%)	14 (37.8%)	23 (62.2%)	0 (0.0%)	0.21
Overweight	157 (63.6%)	81 (51.6%)	71 (45.2%)	5 (3.2%)	
Obese	53 (21.5%)	23 (43.4%)	30 (56.6%)	0 (0.0%)	
Gestational age (weeks)					
6–12	75 (30.4%)	33 (44.0%)	42 (56.0%)	0 (0.0%)	0.18
13–24	92 (37.2%)	48 (52.2%)	40 (43.5%)	4 (4.3%)	
25–38	80 (32.4%)	37 (46.3%)	42 (52.5%)	1 (1.2%)	
Pregnancy order					
First	123 (49.8%)	54 (43.9%)	65 (52.8%)	4 (3.3%)	0.18
Second	107 (43.3%)	57 (53.3%)	50 (46.7%)	0 (0.0%)	
Third	17 (6.9%)	7 (41.2%)	9 (52.9%)	1 (5.9%)	
Trimester					
First	69 (27.9%)	28 (40.6%)	41 (59.4%)	0 (0.0%)	0.14
Second	98 (39.7%)	51 (52.0%)	43 (43.9%)	4 (4.1%)	
Third	80 (32.4%)	39 (48.8%)	40 (50.0%)	1 (1.2%)	

*p value from Chi‐square test.

In terms of antibody concentration, mean IgG levels did not differ significantly between groups for any of the variables examined. The comparison of mean IgG titers according to various study variables is illustrated in Table 3.

**Table 3:** Comparison of mean IgG levels according to study variables (Mann–Whitney and Kruskal–Wallis tests).

Variables	Mean IgG antibody concentration (IU/mL)	Standard deviation (SD)	*p* value* ^,^ **
Age group (years)			
≤ 30	54.19	31.79	0.91*
> 30	53.20	32.22	
Education level			
Illiterate	55.13	32.20	0.48*
Literate	50.40	32.54	
Residence			
Urban	64.86	29.20	0.60*
Rural	60.26	32.97	
Body mass index (BMI)			
Normal	55.88	34.83	0.53**
Overweight	55.38	30.57	
Obese	48.47	34.52	
Gestational age (weeks)			
6–12	55.69	34.64	0.39**
13–24	57.61	30.72	
25–38	48.28	32.15	
Pregnancy order			
First	54.66	32.15	0.97**
Second	53.15	33.63	
Third	51.19	27.63	
Trimester			
First	53.26	32.69	0.22**
Second	60.05	31.87	
Third	47.63	31.69	

* *p* value from Mann–Whitney test

** *p* value from Kruskal–Wallis test.

Due to the limited availability of ELISA kits, IgM testing was performed on 90 participants. Among them, 10 (11.2%) tested positive for IgM, 5 (5.6%) had borderline results, and 75 (83.3%) were negative. Similar to the IgG results, no significant association was found between IgM status and most variables. However, a statistically significant association was observed between pregnancy order and IgM positivity (*p* = 0.006), with higher IgM positivity in women with second or third pregnancies. The association between IgM status and sociodemographic/clinical characteristics is analyzed in Table 4.

**Table 4:** Relationship between IgM status and sociodemographic/clinical characteristics (Chi‐square test).

	IgM antibody against parvovirus B19			
Variables	Negative, *n* (%)	Positive, *n* (%)	Borderline, *n* (%)	*p* value*
Age group (years)				
≤ 30	51 (68%)	5 (6.7%)	2 (2.7%)	0.28
> 30	24 (32%)	5 (6.7%)	3 (4%)	
Education level				
Illiterate	50 (67%)	6 (8%)	5 (7%)	0.31
Literate	25 (33%)	4 (5%)	0 (0%)	
Residence				
Urban	24 (32%)	4 (5%)	2 (3%)	0.73
Rural	51 (68%)	6 (8%)	3 (4%)	
Body mass index (BMI)				
Normal	15 (20%)	1 (1%)	4 (5%)	0.84
Overweight	45 (60%)	6 (8%)	1 (1%)	
Obese	15 (20%)	3 (4%)	0 (0%)	
Gestational age (weeks)				
6–12	27 (36%)	2 (3%)	11 (15%)	0.78
13–24	28 (37%)	4 (5%)	2 (3%)	
25–38	20 (27%)	4 (5%)	2 (3%)	
Pregnancy order				
First	46 (61%)	4 (5%)	1 (1%)	0.006
Second	26 (34%)	5 (7%)	1 (1%)	
Third	3 (4%)	1 (1%)	3 (4%)	
Trimester				
First	24 (32%)	2 (3%)	1 (1%)	0.80
Second	32 (43%)	4 (5%)	2 (3%)	
Third	19 (25%)	4 (5%)	2 (3%)	

*p value from Chi‐square test.

### 3.4 Multivariate Logistic Regression Analysis

Multivariate logistic regression analysis was performed to identify independent predictors of IgG seropositivity. None of the examined variables, including age, education, residence, BMI, gestational age, parity, or trimester, showed a statistically significant association with IgG positivity. Table 5 presents the results of multivariate logistic regression analysis for factors associated with IgG seropositivity.

**Table 5:** Multivariate logistic regression analysis of factors associated with IgG seropositivity.

Variable	Odds ratio (OR)	95% Confidence interval (CI)	*p* value
Age group (years)			
≤ 30	1	—	0.43
> 30	1.83	0.7–41.93	
Education level			
Illiterate	0.39	0.1–13.18	0.09
Literate	1	—	
Residence			
Urban	1	—	0.46
Rural	0.69	0.1–25.87	
Body mass index			
Normal	1	—	0.58
Overweight	0.47	0.2–11.00	0.30
Obese	0.41	0.3–05.29	0.40
Gestational week			
6–12	1	—	0.79
13–24	2.35	0.29–19.05	0.50
25–38	—	—	0.99
Parity			
First	1	—	0.83
Second	1.42	0.5–36.57	0.60
Third	0.95	0.9–09.21	0.90
Trimester			
First	1	—	0.44
Second	0.21	0.2–01.32	0.20
Third	—	—	0.99

## 4 Discussion

Human parvovirus B19 is associated with a broad spectrum of clinical manifestations, the severity and nature of which largely depend on the host's age and immunological status [17]. In pregnant women, infection can have serious consequences such as fetal anemia, spontaneous abortion, and hydrops fetalis [18]. Given the heightened susceptibility of pregnant women and their fetuses to these complications, this study aimed to investigate the seroepidemiology of parvovirus B19 among pregnant women attending Ayatollah Rouhani Hospital in Babol, Iran. The primary finding of this study was that 50% of the participants were seropositive for IgG antibodies against parvovirus B19, indicating past exposure. This rate is comparable to that reported by Mirambo et al. in Mwanza, Tanzania, who found a 55% IgG seroprevalence among 258 pregnant women, and by Sohrabi et al. in Ahvaz, Iran, with a reported rate of 55.7% [14, 19] However, our prevalence was higher than that reported by Karami et al. in Kermanshah, Iran (44.1%) and lower than the 69.1% seropositivity documented by Habibzadeh et al. in Ardabil, Iran [9, 19]. Such discrepancies could be attributed to differences in geographic location, socioeconomic status, climate, healthcare access, and study population characteristics. It is noteworthy that over 40% of our study population lacked IgG antibodies, suggesting susceptibility to primary B19 infection during pregnancy, which can pose serious risks. As noted in Sohrabi et al.'s study, health education and screening programs are crucial in regions with a higher transmission potential, such as southern Iran, due to environmental and hygienic conditions conducive to viral spread [14]. The present study also investigated recent infections by measuring parvovirus B19‐specific IgM antibodies. Among the 90 participants, 11.1% were IgM‐positive, which is considerably higher than the 2% reported by Karami et al. (Kermanshah, Iran), but closer to the 5% reported by Buhtori (Damascus, Syria) [9, 20]. The difference in IgM prevalence across studies is unlikely to be due solely to the transient nature of IgM responses, as all were cross‐sectional in design. Instead, population differences, seasonal variation, and viral circulation levels at the time of sampling likely account for the observed discrepancies. In comparison, a study by Tsujimura et al. using PCR detection among 3000 samples found only one positive case despite a 54% IgG prevalence [21]. This highlights the utility of IgM testing as a feasible and accessible method for identifying recent infections in routine clinical settings. Although PCR is often regarded as a marker of viremia, it can remain positive for several months after acute infection due to the persistence of viral DNA, and thus does not necessarily indicate active replication. Moreover, most patients already exhibit IgM antibodies by the time of clinical presentation. In this context, the primary diagnostic value of PCR lies in its ability to clarify ambiguous serological findings and to rule out false‐positive IgM results, which may occur due to nonspecific reactivity [22]. Therefore, in settings where molecular diagnostics are available, PCR can complement serology to improve diagnostic accuracy. Our IgM data suggest that a portion of our study cohort experienced recent exposure to parvovirus B19, which might have important implications for fetal outcomes. However, it is important to interpret the relatively high IgM seropositivity rate (11.1%) with caution. One explanation may be the natural cyclical pattern of B19V epidemics, which occur every 4–6 years; thus, 2018 might have coincided with a peak transmission year in Iran [23]. Unfortunately, we lacked access to national surveillance data to verify this. An alternative explanation is the possibility of false‐positive IgM results, especially in pregnant women. Serological tests may be affected by nonspecific reactivity or polyclonal B‐cell activation, which are known limitations in this population [24]. Future studies incorporating PCR testing would help clarify these findings. Another strength of this study was the quantification of IgG antibody titers, which allowed a more precise evaluation of immunity levels among pregnant women. Interestingly, our analysis revealed no significant differences in IgG concentrations across various demographic and clinical variables, including age, BMI, gestational age, and trimester. This uniformity may reflect the immunomodulatory adaptations during pregnancy that aim to balance fetal tolerance with effective maternal immune defense, potentially leading to a relatively stable antibody profile despite individual variation. These findings reinforce the importance of measuring antibody levels to assess population‐level vulnerability and guide preventive strategies. We also assessed potential risk factors for B19 seropositivity. In univariate analysis, the number of previous pregnancies showed a significant association with recent B19 infection; however, this did not persist in the multivariate model, possibly due to confounding or collinearity among variables. Interestingly, although higher parity is typically considered a risk factor for B19V due to increased exposure to young children, in our study, the association observed did not follow the expected direction. This inconsistency suggests that the finding in univariate analysis may be due to a chance result, residual confounding, or limited sample size. These findings indicate that parity alone may not be an independent predictor of serostatus once other factors are controlled. Previous studies have explored other predictors. For instance, Mirambo et al. identified a history of low birth weight as a significant factor associated with B19 IgG positivity, and they also noted a decline in antibody titers with increasing gestational age [19]. Although low birth weight was not assessed in our study, unlike the findings of Mirambo et al., we did not observe a significant association between gestational age and IgG seroprevalence; hence, gestational age cannot be considered a predictive factor in our cohort. Understanding the seroepidemiological profile of parvovirus B19 among pregnant women is essential for implementing targeted screening strategies. Identification of IgM‐positive cases could enable timely clinical interventions and monitoring to prevent severe complications such as fetal anemia, miscarriage, and congenital anomalies. The findings of this study support the consideration of routine serological screening during pregnancy, particularly in regions with moderate to high seronegativity and active transmission. Such an approach may help mitigate the potential adverse outcomes associated with parvovirus B19 infection in this vulnerable population. This study had several limitations that should be considered when interpreting the findings. First, real‐time PCR analysis for parvovirus B19 DNA was not performed, which could have provided more definitive evidence of active infections and enhanced the diagnostic accuracy. Second, follow‐up sampling of participants was not feasible, preventing further evaluation of cases with borderline or indeterminate serological results. Third, due to resource limitations, IgM antibody testing could only be performed on a subset of participants, which may have led to an underestimation of the true prevalence of recent or acute B19V infections. Additionally, the sample size was calculated based on the expected seroprevalence of IgG antibodies and was not specifically designed to accurately estimate the incidence of recent infections identified by IgM positivity, which typically necessitates a larger sample due to its lower prevalence. To build upon the findings of this study, future research should incorporate molecular diagnostic methods, such as real‐time PCR, to allow for more accurate identification of active B19 infections. Additionally, follow‐up testing and clinical correlation should be considered to better understand the significance of equivocal serological outcomes. It is also recommended that similar seroepidemiological studies be conducted in other high‐risk populations, including individuals with hemophilia, thalassemia, or those receiving dialysis, to more comprehensively assess the burden of parvovirus B19 among immunocompromised and transfusion‐dependent groups.

## 5 Conclusion

This study demonstrated that approximately half of the pregnant women attending Ayatollah Rouhani Hospital in Babol had prior exposure to human parvovirus B19, as indicated by IgG seropositivity. Moreover, 11.1% of participants exhibited IgM antibodies, suggesting recent infection. These findings highlight a substantial portion of the population lacking immunity and potentially at risk for primary infection during pregnancy, which could lead to adverse fetal outcomes. The overall seroprevalence rates observed are generally in line with those reported in similar studies across Iran and other countries.

## Author Contributions

F.S. and M.B. conceptualized and designed the study. F.S., S.E., H.G., M.J., and M.G. analyzed and interpreted the data. Z.D., A.E.S., and A.M. drafted the manuscript. All authors reviewed and approved the final version of the manuscript. All authors have read and approved the final version of the manuscript. The corresponding author had full access to all of the data in this study and takes complete responsibility for the integrity of the data and the accuracy of the data analysis.

## Conflicts of Interest

The authors declare no conflicts of interest.

## Transparency Statement

The lead authors, Farzin Sadeghi and Masomeh Bayani, affirm that this manuscript is an honest, accurate, and transparent account of the study being reported; that no important aspects of the study have been omitted; and that any discrepancies from the study as planned (and, if relevant, registered) have been explained.

## Data Availability

The data supporting the findings of this study are available from the corresponding author upon reasonable request.
